# Perceptions of Fall Prevention and Engagement in Social Prescribing Activities Among Older Adults: A Cross-Sectional Study in Portugal

**DOI:** 10.3390/healthcare13243209

**Published:** 2025-12-08

**Authors:** Cristiano Matos, Cristina Rosa Baixinho, Violeta Alarcão, Maria Adriana Henriques, Ricardo Oliveira Ferreira, Tiago Nascimento, Miguel Arriaga, Tatiana Alves, Paulo Nogueira, Andreia Costa

**Affiliations:** 1 Instituto de Saúde Ambiental (ISAMB), Faculdade de Medicina, Universidade de Lisboa, 1600-190 Lisboa, Portugalvioleta_sabina_alarcao@iscte-iul.pt (V.A.); ahenriques@esel.pt (M.A.H.); rf@esel.pt (R.O.F.); tnascimento@esel.pt (T.N.); miguelarriaga@dgs.min-saude.pt (M.A.); pnogueira@medicina.ulisboa.pt (P.N.); 2 Nursing Research, Innovation and Development Centre of Lisbon (CIDNUR), Nursing School of Lisbon (Escola Superior de Enfermagem de Lisboa), University of Lisbon, 1600-190 Lisboa, Portugal; crbaixinho@esel.pt; 3 Center for Innovative Care and Health Technology (ciTechcare), 2414-016 Leiria, Portugal; 4 Centro de Investigação e Estudos de Sociologia (CIES-ISCTE), Instituto Universitário de Lisboa (ISCTE), 1649-026 Lisboa, Portugal; 5 Laboratório Associado TERRA, Faculdade de Medicina, Universidade de Lisboa, 1649-028 Lisboa, Portugal; 6 Católica Research Centre for Psychological—Family and Social Wellbeing (CRC-W), Faculdade de Ciências Humanas, Universidade Católica Portuguesa, 1649-023 Lisboa, Portugal; 7 National Institute of Health Dr. Ricardo Jorge, Epidemiology Department, 1649-016 Lisboa, Portugal; tatiana.alves@insa.min-saude.pt; 8 Instituto Politécnico de Setúbal, Escola Superior de Saúde, 2910-761 Setúbal, Portugal; 9 Instituto de Ciências Sociais, Universidade de Lisboa, 1600-189 Lisboa, Portugal

**Keywords:** fall prevention, older adults, social prescribing, healthy ageing

## Abstract

**Background**: Falls are a major cause of injury, functional decline, and reduced quality of life among older adults, posing a significant public health challenge. Social prescribing is gaining relevance in gerontology, offering structured strategies to engage individuals in preventive activities, including fall prevention strategies, through engagement in community-based activities. **Aim**: To examine older adults’ perception of the relevance of personal protection and development activities (e.g., prevention against falling) and compare sociodemographic, behavioural, and engagement profiles between those who agree and those who disagree with its relevance. **Methods**: A cross-sectional study was conducted with 613 older adults aged 65–93 years. Data collection included sociodemographic, health-related, and behavioural/social engagement variables (including perceptions regarding the benefits of social prescribing and interest in community-based activities). For this analysis, participants were dichotomized based on their agreement with the relevance of personal protection and development activities (e.g., prevention against falling). Of the 569 participants included, 538 (94.5%) agreed with its relevance and 31 (5.5%) disagreed. Descriptive and exploratory analyses were conducted to compare the two groups across variables. Multivariate logistic regression analyses were conducted to explore independent predictors of agreement across sociodemographic, behavioural, social prescribing, and health-related variables. **Results**: Significant differences were observed between the groups in awareness of active ageing (*p* = 0.018), volunteering (*p* < 0.001), participation in social and community activities (*p* < 0.001), and hobbies like gardening, fishing, or cooking (*p* = 0.002). Those who agreed with the importance of personal protection and development activities were significantly more likely to value a range of initiatives, including social activities in recreational organizations, physical activity in the community (e.g., hiking), artistic and creative activities (e.g., visual arts, music), technical or technological activities (e.g., do-it-yourself, computers), and cultural enrichment activities (e.g., visiting museums), (*p* < 0.001). Multivariate analyses showed no effects of sociodemographic or health-related factors, whereas behavioural and engagement-related variables—including volunteering, hobbies, and several social prescribing activities—significantly predicted agreement with the relevance of personal protection and development activities. Discussion: The findings suggest that older adults who perceive fall prevention as relevant are more actively engaged in diverse health-promoting activities, including volunteering, hobbies, and community-based programmes. This pattern may reflect higher health literacy, stronger social networks, and proactive attitudes towards ageing. **Conclusions**: Perceptions of fall prevention are closely linked to broader patterns of engagement in health-promoting activities among older adults. Recognizing and addressing differences in how these activities are valued can inform more inclusive and targeted gerontological interventions.

## 1. Introduction

Falls are widely recognized as one of the most pressing public health problems affecting older adults [1], being a leading cause of injury and hospitalization, but also a determinant of disability, loss of independence, and premature mortality [1,2,3,4]. The consequences of falls extend far beyond the individual, with a substantial impact on families, caregivers, and healthcare systems, given the costs associated with emergency services, rehabilitation, and long-term care [5,6]. Furthermore, the psychological consequences, such as fear of falling and subsequent restriction of activities, contribute to functional decline, social isolation, and diminished quality of life [7].

The ageing of the population intensifies this challenge, with the proportion of older patients rising in recent decades [8,9]. Many of these individuals live with frailty, multimorbidity, or other age-related vulnerabilities, which increase the risk of falls [3,9]. Thus, fall prevention has emerged as a priority for promoting healthy ageing and ensuring the sustainability of health and social care systems [10,11], since it represents a considerable burden, generating high costs associated with emergency care, hospital admissions, rehabilitation, and long-term institutionalization, in addition to increasing demands on caregivers and community services [12,13].

The conventional fall prevention approaches have largely centred on clinical interventions, such as balance training, medication reviews, and home modifications, often applied after a first fall has occurred [14,15,16]. While effective to some extent, such approaches may overlook the broader determinants of falls, which include physical status but also psychosocial and environmental factors. Evidence increasingly supports the need for multidimensional interventions that go beyond the healthcare setting to embrace lifestyle, social participation, and community engagement as key protective factors [17,18].

In this context, social prescribing could be seen as an innovative model of care in primary health [19,20], which enables healthcare professionals to refer individuals to non-clinical services and community-based resources—including cultural, recreational, educational, and physical activities—aimed at addressing social determinants of health and enhancing well-being [20], promoting active engagement in different activities, and foster social connectedness, combat loneliness, and supports autonomy in later life [21,22]. These outcomes can be highly relevant for fall prevention, as stronger social networks, higher levels of physical activity, and improved Health Literacy (HL) have all been associated with reduced fall risk [23,24,25].

Recent studies have started to examine older adults’ perceptions of social prescribing; Costa et al. (2024) conducted a national survey in Portugal showing high acceptance of social prescribing among older adults and identifying “personal protection and development” and “cultural enrichment” activities as particularly valued domains [19]. However, that study did not focus on fall prevention as a specific component within these activities, nor did it compare groups of older adults who agreed or disagreed with its relevance. In parallel, Percival et al. (2022) conducted a systematic review that concluded that, despite increasing recognition of social prescribing as a tool to enhance well-being in later life, there remains a lack of empirical studies exploring the mechanisms through which engagement in non-clinical activities translates into preventive outcomes [26].

Despite this, the integration of fall prevention strategies into social prescribing frameworks remains limited and insufficiently explored in research: little is known about how older adults themselves perceive the relevance of activities related to personal protection and development, such as fall prevention programmes, within the broader scope of community and social interventions.

The interplay between social capital, active ageing, and health literacy offers a valuable lens to interpret how older adults engage with preventive health initiatives. Social capital, through networks of trust, reciprocity, and social participation, creates the structural context in which individuals can access resources and support for healthy behaviours. Active ageing represents the functional outcome of this process, encompassing continued participation, autonomy, and self-fulfilment. Health literacy, in turn, acts as a mediating mechanism that enables older adults to interpret health information, make informed decisions, and translate social engagement into preventive action. Conceptually, these constructs are mutually reinforcing: higher health literacy fosters the capacity to mobilize social capital; social participation promotes active ageing; and engagement in active ageing behaviours further strengthens literacy and social connectedness.

The present study seeks to characterize the perceptions of older adults regarding the relevance of personal protection and development activities, with a particular focus on fall prevention. Specifically, it compares two groups—those who agree and those who disagree with the importance of such activities—across sociodemographic, health-related, and behavioural/social engagement variables (including social prescribing-related dimensions).

## 2. Materials and Methods

### 2.1. Study Design and Participants

The study employed a cross-sectional design with a representative sample of community-dwelling older adults in mainland Portugal. Data were collected between September and October 2022 through structured telephone interviews, conducted by trained interviewers using computer-assisted techniques. Participants were randomly selected from a pool of landline and mobile phone numbers generated by a specialized polling organization. The minimum required sample size was calculated using the OpenEpi tool (Version 3.01), assuming a 95% confidence level and a 5% margin of error. Considering a large population base of more than one million individuals and an assumed prevalence of 50%, which ensures maximum variability, the estimated minimum sample size was 384 participants. This calculation was supported by data from the most recent national census, indicating that approximately 2.3 million Portuguese residents are aged 65 years or older.

Eligibility criteria included being aged 65 years or older and living in mainland Portugal. Of those contacted, 613 individuals completed the survey, yielding a response rate of 74%. The structured questionnaire, developed by the research team based on prior studies and validated instruments on ageing and social prescribing [19], gathered information on sociodemographic (e.g., age, sex, marital status, education, employment situation), economic (e.g., perceived household financial situation), and health-related variables (e.g., presence of chronic conditions or disabilities, self-rated health).

The EQ-5D-3L instrument was used to assess health-related quality of life. It consists of a brief questionnaire and a visual analogue scale (VAS). The questionnaire includes five dimensions—mobility, self-care, usual activities, pain/discomfort, and anxiety/depression—each rated on a three-level scale (1 = no problems, 2 = some problems, 3 = extreme problems) [27]. It also assessed participants’ perceptions of the benefits of social prescribing in terms of health and well-being, community cohesion, quality of care, and primary care functioning, and the usefulness of several categories of social prescribing activities (e.g., cultural, recreational, physical, artistic, technical, touristic, and personal protection/development activities), using a 7-point scale (1 = completely disagree to 7 = completely agree).

For this study, the variable “*usefulness of social prescribing activities related to personal protection and development activities (as prevention against falling)*” was used to dichotomize the sample into two groups: those who agreed with its relevance and those who disagreed. Individuals who selected a neutral response were excluded (n = 44), resulting in a final analytical sample of 569 older adults. Among these, 538 (94.5%) agreed with the relevance of personal protection and development activities, while 31 participants (5.5%) disagreed.

### 2.2. Ethical Considerations

All participants provided oral informed consent prior to participation. The study protocol was approved by the Ethics Committee of the Centro Académico de Medicina de Lisboa (Process no. 193/22) and adhered to the principles of the Declaration of Helsinki and the General Data Protection Regulation (GDPR).

### 2.3. Statistical Analysis

Data were analyzed using IBM SPSS Statistics 27.0 (IBM Corp., Armonk, NY, USA). Descriptive statistics, including frequencies, means, medians, standard deviations, and interquartile ranges, were used to characterize the sample. Group comparisons (agree vs. disagree) were conducted using Chi-square or Fisher’s exact tests for categorical variables, depending on the distribution of cell frequencies. For continuous variables, data distributions were assessed through histograms, Q–Q plots, and the Shapiro–Wilk test. Variables meeting normality assumptions were compared using independent samples *t*-tests, while the Mann–Whitney U test was applied to non-normally distributed variables. Statistical significance was set at *p* < 0.05. Effect sizes were estimated using Cohen’s *d* and eta squared (η^2^). For analytical purposes, the Likert scale was treated as a continuous variable to allow calculation of descriptive statistics and group comparisons [28].

Although the unequal size of the agreement groups limited the stability of multivariate modelling, exploratory adjusted analyses were conducted using binary logistic regression to examine potential predictors of agreement with the relevance of personal protection and development activities.

## 3. Results

Sociodemographic characteristics of the sample are presented in Table 1. The majority of participants were aged between 65 and 74 years, married, and not working professionally. Educational levels were heterogeneous, with a predominance of basic and secondary education. Most respondents rated their household’s financial situation as sufficient for their needs and their overall health as acceptable.

When comparing the two groups—those who agreed versus those who disagreed with the relevance of personal protection and development activities—no statistically significant differences were found across sociodemographic variables, employment status, perceived financial situation, or self-rated health (all *p* > 0.05). This indicates that perceptions regarding the importance of fall prevention were not explained by basic demographic or economic characteristics.

Table 2 shows that most factors were perceived as contributing to active ageing, with consistently high mean scores across groups. Statistically significant differences were observed for volunteer activities (*p* < 0.001), social and community participation (*p* < 0.001), having hobbies (*p* = 0.008), and saving sufficient money for retirement (*p* = 0.014), which were rated higher contributor by the group that consider social prescribing activities of personal protection and development activities (as prevention against falling) as useful. No significant group differences were observed for health-related factors such as physical activity, diet, oral health, or mental health.

In Table 3, the perceived relevance of different social prescribing activities by group is represented. All activities were rated significantly higher by the group that considered social prescribing activities of personal protection and development activities as useful (*p* < 0.001 for all comparisons).

Quality of life scores assessed by the EQ-5D-3L instrument for both groups are shown in Table 4. Perceived health state expressed with Index and VAS were very similar between groups, with no significant differences (*p* = 0.981 and *p* = 0.659).

Across the five EQ-5D dimensions—mobility, self-care, usual activities, pain/discomfort, and anxiety/depression—both groups presented comparable distributions. The majority of participants reported no problems with mobility, self-care, and usual activities, although a considerable proportion indicated some difficulties with pain/discomfort and anxiety/depression. No statistically significant differences emerged between groups in any of these dimensions (all *p* > 0.05).

The logistic regression assessing whether sociodemographic characteristics predicted agreement with the relevance of personal protection and development activities (including fall prevention) showed that none of the variables were significant predictors of the outcome: sex (*p* = 0.677), age (*p* = 0.575), marital status (*p* = 0.875), educational level (*p* = 0.195), employment status (*p* = 0.827), perceived financial situation (*p* = 0.627), and self-rated health (*p* = 0.842). The logistic regression model did not improve upon the null model, suggesting that sociodemographic factors do not account for the differences in perceptions observed. This is consistent with the univariate findings and supports the interpretation that attitudes toward fall prevention are not shaped by basic demographic or economic characteristics.

Regarding perceived factors contributing to active ageing, the logistic regression revealed three contributors to active ageing that independently predicted agreement with the relevance of personal protection and development activities. Perceiving volunteering as important was the strongest predictor (OR = 4.15, *p* < 0.001), followed by having hobbies such as gardening, fishing, or cooking (OR = 2.46, *p* = 0.015). Regular physical activity showed a modest negative association (OR = 0.31, *p* = 0.006). All other perceived contributors to active ageing were non-significant and did not enter the model. The final logistic regression model explained approximately 12.1% according to the Nagelkerke R^2^, indicating a small explanatory capacity for the predictors included. The logistic regression model explained approximately 12.1% according to the Nagelkerke R^2^, indicating a small explanatory capacity for the predictors included.

In the perceived relevance of different social prescribing activities, the logistic regression identified four activity domains that predicted agreement with the relevance of personal protection and development activities. Social activities in recreational associations emerged as a significant predictor (OR = 1.62, *p* < 0.001), indicating that individuals who value social participation are more likely to recognize the importance of fall-prevention-related initiatives. Technical or technological activities, such as do-it-yourself or computer use also showed a positive association (OR = 1.35, *p* = 0.031), suggesting that engagement in practical or skill-building tasks aligns with valuing preventive approaches. Cultural enrichment activities, including museum or monument visits, demonstrated a smaller but noteworthy effect (OR = 1.28, *p* = 0.080), while touristic activities such as excursions remained a strong and consistent predictor across steps (OR = 1.67, *p* = 0.001). All other activity domains were not retained in the final model. The final logistic regression model explained approximately 52.2% according to the Nagelkerke R^2^, indicating a moderate to strong explanatory capacity for the predictors included.

Regarding quality-of-life variables assessed through the EQ-5D-3L, the logistic regression analysis showed that none of the dimensions—mobility, self-care, usual activities, pain/discomfort, or anxiety/depression—significantly predicted agreement with the relevance of personal protection and development activities (all *p* > 0.20). In addition, neither perceived health state (EQ-5D index score) nor self-rated health status (EQ-5D VAS score) showed any meaningful association with the outcome, indicating that older adults’ agreement levels were not influenced by their general health perception or subjective health evaluation. Overall, these findings suggest that perceptions of the importance of personal protection and fall-prevention activities are not shaped by self-reported health status, functional ability, or perceived health quality, but instead appear to be driven by behavioural and engagement-related factors.

## 4. Discussion

The older adults’ perceptions of the relevance of personal protection and development activities, such as fall prevention, were examined and compared between individuals who agreed with their importance to those who did not. Significant differences were observed across behavioural and social engagement variables, including awareness of active ageing, volunteering, participation in social and community activities, and involvement in hobbies such as gardening, fishing, or cooking. Moreover, older adults who valued fall prevention were more likely to recognize the usefulness of a wide range of social prescribing initiatives, including recreational, physical, artistic, technological, and cultural activities.

These results suggest that perceptions of fall prevention are closely intertwined with broader health-promoting behaviours and attitudes. Older adults who acknowledge the importance of preventing falls appear to adopt a more proactive approach to ageing, reflected in their greater social participation and community engagement. This pattern is consistent with evidence linking health literacy and positive attitudes toward ageing with increased adherence to preventive behaviours and improved health outcomes [24]. Participants who agreed with the importance of fall prevention rated certain active ageing factors—such as volunteering, social participation, and hobbies—more positively than those who did not consider fall prevention relevant. This consistent pattern suggests that perceiving fall prevention as important may reflect a broader understanding of “active ageing,” encompassing autonomy, engagement, and personal fulfilment. For these individuals, preventive strategies appear to be viewed not as restrictive or risk-focused, but rather as enabling continued participation, independence, and well-being in later life. These findings underscore the importance of framing prevention as a component of active and healthy ageing, rather than solely as a response to vulnerability or risk.

Beyond conceptual alignment, several psychosocial and cultural factors may explain why some older adults place less emphasis on fall prevention. Previous studies have shown that acknowledging the risk of falling can be perceived as an admission of frailty, dependence, or ageing itself—conditions often stigmatized in many cultures [29,30]. As a result, some individuals may distance themselves from preventive messages to preserve a sense of autonomy and invulnerability. This response aligns with the notion of “positive denial,” whereby older adults prefer to identify as active and self-reliant rather than at risk. Moreover, cultural norms that emphasize independence and productivity in later life may further discourage engagement in interventions framed explicitly around vulnerability or risk reduction.

A similar pattern was observed in the assessment of social prescribing activities (Table 3), where those who considered fall prevention relevant also attributed greater usefulness to all other types of community-based activities. This consistent association suggests that older adults who value fall prevention tend to recognize broader benefits in participating in social, cultural, and recreational initiatives. For these individuals, engagement in such activities may represent both an enjoyable and purposeful means of maintaining autonomy, social connection, and overall well-being. This pattern highlights that preventive and health-promoting motivations are not mutually exclusive but rather intertwined within a holistic understanding of active ageing. Consequently, communication strategies should frame fall-prevention initiatives as empowering, enriching, and socially meaningful experiences that support independence and participation, rather than as narrowly corrective or risk-avoidance measures.

The association between valuing fall prevention and involvement in volunteering and community-based activities highlights the role that social capital can have in shaping health behaviours [31]. Some studies have emphasized the importance of education and social engagement in fall prevention, highlighting that preventive education for older adults and their caregivers is critical and that social participation enhances the effectiveness of interventions. Educational strategies increase awareness of risk factors, improve self-management skills, and foster confidence in adopting preventive behaviours, while social engagement provides motivation, peer support, and opportunities to integrate these behaviours into daily life. Evidence suggests that when educational initiatives are combined with community participation, they not only improve knowledge but also translate into greater adherence to preventive practices and sustained reductions in fall risk [32,33]. Social prescribing aims to strengthen these networks by facilitating access to meaningful activities that promote autonomy, reduce isolation, and support functional capacity in later life [26,34]. Our findings suggest that individuals who already recognize the importance of fall prevention may be more receptive to such interventions, while those who dismiss their relevance could represent a harder-to-reach subgroup at greater risk.

From a theoretical perspective, according to the Transtheoretical Model, individuals who do not recognize the relevance of fall prevention may be in the precontemplation stage, characterized by low awareness of personal risk and limited readiness to adopt preventive behaviours [35]. Similarly, within the framework of the Health Belief Model (Rosenstock, 1974), these individuals may perceive low susceptibility to falls or low perceived benefits of preventive action, which in turn reduces their motivation to engage in fall-prevention initiatives [36]. Understanding this stage of readiness and perception of risk can help tailor interventions, for instance, by emphasizing positive outcomes such as autonomy and confidence rather than focusing exclusively on risk reduction.

Tailored strategies are therefore needed to engage this population, for example, through targeted communication campaigns, peer-led initiatives, or low-threshold community programmes. Community-based programmes, such as the STEADI initiative, illustrate the potential of combining multifactorial risk assessments with educational and community resources, showing effectiveness in reducing fall-related hospitalizations [37]. This type of collaboration resonates with the principles of social prescribing and reinforces its applicability in fall prevention [37]. Although no significant differences were found between groups regarding mobility levels, the descriptive patterns suggest that individuals with lower mobility may nonetheless express openness to cultural, recreational, and even physical activities within the community [38], indicating a willingness to maintain autonomy and social participation, provided that activities are adapted to functional capacity and accessible in their format. Future studies should examine in greater depth how mobility limitations interact with perceptions of social prescribing and identify the types of activities most valued by those with restricted physical function.

The multivariate findings reinforce that perceptions of the relevance of personal protection and development activities are not shaped by sociodemographic or health-related factors, but rather by behavioural and engagement-related characteristics. Older adults who value volunteering, hobbies, and a range of social prescribing activities were significantly more likely to recognize the importance of these preventive initiatives, suggesting that proactive engagement in community and leisure activities reflects a broader orientation toward active and autonomous ageing. In contrast, self-rated health, functional limitations and demographic attributes showed no meaningful influence, indicating that attitudes toward fall prevention arise less from objective health status and more from lifestyle patterns, interests, and existing social connectedness. This highlights the importance of integrating fall-prevention messages within socially meaningful and engaging contexts rather than relying solely on traditional risk-based communication.

The results also highlight the multidimensional nature of falls, which emphasizes that prevention must go beyond risk factors to encompass physical, social, and environmental determinants [8,39]. Participants who valued fall prevention also expressed greater interest in cultural, creative, and technological activities, suggesting that preventive behaviours are embedded in a wider lifestyle that promotes cognitive stimulation, social connectedness, and active ageing. However, participation in preventive programmes is often limited by individual barriers, such as denial or fear of being a burden, as well as community-level constraints, including transportation difficulties and limited resources [40]. Recognizing and addressing these obstacles is essential, since overcoming them directly supports the World Health Organization’s definition of healthy ageing, which places functional ability and active participation in society at the core of ageing policies [41]. Future studies should associate falls and fear of falling with activities performed associated with social prescription.

Another key implication concerns health literacy: the differences observed between groups may reflect varying levels of knowledge, awareness, and confidence in managing health risks. Low health literacy has been linked to reduced engagement in preventive care, poorer self-management, and higher vulnerability to adverse outcomes, including falls [24,42]. A review that aimed to explore and synthesize the current evidence regarding the role of HL in enhancing fall prevention in hospital and community settings concludes that HL can be increased through tailored information, verbal debriefing, interactive communication, and culturally adapted interventions. Low HL impedes the understanding of education, engaging interventions, and using technological devices in implementing fall-prevention interventions [24]. Therefore, interventions to promote fall prevention within social prescribing should not only provide access to activities but also address underlying literacy gaps, empowering older adults to recognize the value of preventive behaviours. Thus, combining fall-prevention education with accessible community-based opportunities may represent a dual pathway to strengthen health literacy and social capital simultaneously.

The results suggest that social prescribing should be integrated into nursing care as a person-centred strategy to promote physical activity, prevent social isolation, and address key risk factors for falls. This approach also supports health literacy and facilitates referrals to appropriate community resources. By incorporating social prescribing into their practice, nurses can broaden fall-prevention interventions, enhance patient engagement, strengthen local support networks, and ultimately contribute to the preservation of autonomy and quality of life.

Finally, the findings have implications for health and social policy. In contexts where healthcare systems remain focused on secondary care, integrating fall prevention into primary care and community-based services is critical [43,44]. Social prescribing can serve as a bridge between health and social sectors, offering scalable and sustainable strategies to mitigate fall risk and support functional independence. Ensuring equitable access and tailoring interventions to those less convinced of their relevance will be essential to maximize impact.

### Limitations

This study has some limitations. First, because this study used a cross-sectional design, it can only identify associations and not establish causal relationships between perceptions of fall prevention and engagement in health-promoting activities. It is therefore not possible to determine whether valuing fall prevention leads to greater participation in such activities, whether participation fosters greater awareness of fall prevention, or whether both are influenced by other underlying factors. Second, the small size of the “disagree” group limits statistical power for some analyses and may reduce the generalizability of subgroup comparisons. The authors acknowledge that future studies with larger samples could benefit from a multivariate logistic regression model to further assess independent predictors of agreement on the usefulness of fall prevention. In addition, self-reported data are subject to recall and social desirability biases. The study did not include objective data on participants’ fall history or clinical risk of falling, which limits the ability to contextualize perceptions of fall prevention in relation to actual experiences or needs. Future studies should explore longitudinal associations between perceptions of fall prevention and actual participation in social prescribing activities, as well as evaluate interventions designed to shift perceptions among those less convinced of their relevance. Additionally, qualitative research could provide richer insights into the reasons why some older adults undervalue fall prevention, helping to design tailored and culturally sensitive approaches. A key strength of this study lies in its large and diverse sample of older adults, which allowed for meaningful comparisons across sociodemographic and behavioural variables. The inclusion of a wide range of activity types also provides a nuanced understanding of how perceptions of fall prevention relate to broader patterns of engagement in health-promoting behaviours.

## 5. Conclusions

This study highlights that older adults who recognize the importance of personal protection and development activities, such as fall prevention, are more actively engaged in a wide range of health-promoting initiatives, including volunteering, hobbies, and community-based programmes. These findings suggest that perceptions of fall prevention are not isolated but integrated into broader patterns of proactive ageing, supported by higher health literacy and stronger social networks.

To maximize the potential of social prescribing in gerontology, it is crucial to raise awareness about the value of preventive activities among those less inclined to recognize their relevance. Multifactorial interventions, exercise programmes, and community engagement are all critical components of fall prevention that can be facilitated through social prescribing. Tailored strategies that enhance health literacy, expand access to community resources, and foster inclusive participation may contribute to reducing fall risk, improving quality of life, and supporting healthy ageing at a population level. Further research is needed to explicitly evaluate the impact of social prescribing on fall prevention outcomes among older adults.

The findings suggest that communication about fall prevention should be reframed to emphasize autonomy, confidence, and continued participation rather than vulnerability or risk. Health professionals—particularly nurses and primary care teams—can integrate motivational approaches and person-centred dialogue to align preventive messages with older adults’ values and self-perceptions. Community programmes may also benefit from embedding fall-prevention activities within broader social prescribing initiatives, such as cultural or recreational events, to reduce stigma and increase engagement. Tailoring materials to different levels of health literacy, using peer-led communication, and presenting prevention as a way to “stay active and independent” rather than “avoid falling” may further enhance participation and adherence.

## Figures and Tables

**Table 1:** Sociodemographic Characteristics.

Variable	Category	Agree (n = 538)	Disagree (n = 31)	*p*-Value
Sex	Male	281	15	Fisher’s exact test: 0.715
	Female	257	0	
Age, years	65–74	355	23	Chi-square test 0.088
	75–84	163	5	
	>84	20	3	
Marital status	Married	349	17	Chi-square test: 0.347
	Other	189	14	
Educational level	No schooling or First cycle of basic education (4th grade)	148	6	Chi-square test 0.597
	Second or third cycle of basic education (6th–9th grade)	110	5	
	Secondary education (12th grade)	145	10	
	Bachelor’s/Master’s/PhD degree	135	10	
Employment situation	Working professionally	36	1	Fisher’s exact test: 0.712
	Not working professionally	501	30	
Perceived household financial situation	Comfortable or very comfortable	150	9	Chi-square test 0.640
	Enough for needs	231	11	
	Difficult or very difficult	153	11	
Overall health rating	Good/Very good	168	10	Chi-square test 0.965
	Acceptable	273	15	
	Bad/Very bad	97	6	

**Table 2:** Perceived Factors Contributing to Active Ageing.

“In Your Opinion, Which Factors Contribute to Ageing Actively?”	Mean (SD) Agree	Mean (SD) Disagree	Difference *p*-Value *t*-Test	Effect Size (Cohen’s *d*)
Being free from chronic diseases such as diabetes or heart disease	3.08 (0.66)	3.23 (0.81)	0.143	
Being able to walk independently, even with physical limitations	3.15 (0.64)	3.26 (0.68)	0.343	
Engaging in regular physical activity or exercise	3.26 (0.54)	3.26 (0.58)	0.960	
Eating a healthy and balanced diet	3.33 (0.51)	3.16 (0.64)	0.179	
Avoiding the consumption of alcoholic beverages	3.00 (0.69)	2.90 (0.70)	0.505	
Maintaining good oral health	3.32 (0.50)	3.19 (0.60)	0.303	
Having good mental health	3.42 (0.51)	3.26 (0.63)	0.185	
Not experiencing loneliness	3.29 (0.61)	3.13 (0.72)	0.233	
Participating in volunteer activities	3.22 (0.55)	2.84 (0.64)	<0.001	Medium 0.64
Participating in social and community activities	3.24 (0.52)	2.90 (0.54)	<0.001	Medium 0.64
Having hobbies such as gardening, fishing, or cooking	3.31 (0.54)	3.00 (0.68)	0.008	
Continuing to learn new things	3.37 (0.51)	3.26 (0.51)	0.240	
Saving sufficient money for retirement	3.22 (0.58)	2.90 (0.70)	0.014	
Having adequate housing	3.35 (0.51)	3.13 (0.72)	0.113	

Responses were collected on a 7-point Likert scale. For analytical purposes, the Likert scale was treated as a continuous variable to allow calculation of means and group comparisons.

**Table 3:** Relevance of different social prescribing activities by group—differences and correlation.

Relevance of Different Social Prescribing Activities by Group	Median—IQR Agree	Median—IQR Disagree	Difference *p*-Value Mann–Whitney	Effect Size (eta Square)
Cultural enrichment (e.g., visiting museums)	6.16 (0.91)	3.97 (2.12)	<0.001	Small η^2^ = 0.017
Recreational/social activities	5.63 (1.34)	2.97 (1.64)	<0.001	Small η^2^ = 0.018
Physical activity in community (e.g., hiking)	5.90 (1.22)	3.65 (2.09)	<0.001	Small η^2^ = 0.014
Artistic/creative activities (e.g., music, visual arts)	5.65 (1.36)	3.23 (1.73)	<0.001	Small η^2^ = 0.016
Technical/technological activities (e.g., DIY, computers)	5.80 (1.30)	3.29 (1.67)	<0.001	Small η^2^ = 0.019
Touristic activities (e.g., excursions)	5.71 (1.37)	2.77 (1.67)	<0.001	Small η^2^ = 0.019

Responses were collected on a 7-point Likert scale. For analytical purposes, the Likert scale was treated as a continuous variable to allow calculation of means and group comparisons.

**Table 4:** Quality Life Scores.

Dimension		Agree (n = 538)	Disagree (n = 31)	*p*-Value
Perceived health state (index) Mean (SD)		0.83 (0.20)	0.82 (0.18)	*t*-test *p* = 0.981
Perceived health status (VAS) Mean (SD)		70.65 (23.48)	72.54 (20.98)	*t*-test *p* = 0.659
Mobility	No problems	339 (63.01%)	23 (74.19%)	Fisher exact *p* = 0.252
	Some/Extreme problems	199 (36.98%)	8 (25.81%)	
Self-care	No problems	502 (93.31%)	29 (93.55%)	Fisher exact *p* = 1.000
	Some/Extreme problems	36 (6.68%)	2 (6.45%)	
Usual activities	No problems	420 (78.07%)	24 (77.42%)	Chi-square *p* = 1.000
	Some/Extreme problems	118 (21.93%)	7 (22.58%)	
Pain/discomfort	No problems	239 (44.43%)	12 (38.71%)	Chi-square *p* = 0.581
	Some/Extreme problems	299 (55.58%)	19 (61.29%)	
Anxiety/depression	No problems	363 (67.47%)	18 (58.06%)	Chi-square *p* = 0.427
	Some/Extreme problems	175 (32.52%)	12 (41.94%)	

## Data Availability

The data presented in this study are available on reasonable request from the corresponding author due to confidentiality and privacy restrictions. In accordance with the informed consent provided to participants, the responses are stored securely, accessible only to the research team, and will be deleted five years after publication of the articles resulting from the study, in compliance with applicable data protection legislation.

## References

[1] Vaishya R., Vaish A. (2020). Falls in older adults are serious. Indian J. Orthop..

[2] Malmivaara A., Heliövaara M., Knekt P., Reunanen A., Aromaa A. (1993). Risk factors for injurious falls leading to hospitalization or death in a cohort of 19,500 adults. Am. J. Epidemiol..

[3] Pitchai P., Dedhia H.B., Bhandari N., Krishnan D., D’Souza N.R.J., Bellara J.M. (2019). Prevalence, risk factors, circumstances for falls and level of functional independence among geriatric population-A descriptive study. Indian J. Public Health.

[4] Toyabe S. (2010). World Health Organization fracture risk assessment tool in the assessment of fractures after falls in hospital. BMC Health Serv. Res..

[5] Tzeng H.-M., Yin C.-Y. (2009). Inpatient falls: The impact of family and personal caregivers. Appl. Nurs. Res..

[6] Faes M.C., Reelick M.F., Joosten-Weyn Banningh L.W., Gier Mde Esselink R.A., Olde Rikkert M.G. (2010). Qualitative study on the impact of falling in frail older persons and family caregivers: Foundations for an intervention to prevent falls. Aging Ment. Health.

[7] Jørstad E.C., Hauer K., Becker C., Lamb S.E., Group P. (2005). Measuring the psychological outcomes of falling: A systematic review. J. Am. Geriatr. Soc..

[8] World Health Organization (2021). Falls [Internet].

[9] Peel N.M. (2011). Epidemiology of falls in older age. Can. J. Aging/La Rev. Can. Du Vieil..

[10] Ungar A., Rafanelli M., Iacomelli I., Brunetti M.A., Ceccofiglio A., Tesi F., Marchionni N. (2013). Fall prevention in the elderly. Clin. Cases Miner. Bone Metab..

[11] Pfortmueller C.A., Lindner G., Exadaktylos A.K. (2014). Reducing fall risk in the elderly: Risk factors and fall prevention, a systematic review. Minerva Med..

[12] James S.L., Lucchesi L.R., Bisignano C., Castle C.D., Dingels Z.V., Fox J.T., Hamilton E.B., Henry N.J., Krohn K.J., Liu Z. (2020). The global burden of falls: Global, regional and national estimates of morbidity and mortality from the Global Burden of Disease Study 2017. Inj. Prev..

[13] Cotter P.E., Timmons S., O’Connor M., Twomey C., O’Mahony D. (2006). The financial implications of falls in older people for an acute hospital. Ir. J. Med. Sci..

[14] Noohu M.M., Dey A.B., Hussain M.E. (2014). Relevance of balance measurement tools and balance training for fall prevention in older adults. J. Clin. Gerontol. Geriatr..

[15] Suen J., Narayan S., Seppala L.J., van der Velde N., Sherrington C., Sutcliffe K., Cameron I.D., Kneale D., Dyer S.M. (2025). Features of successful medication review and deprescribing interventions for fall prevention in residential aged care facilities: An intervention component analysis of an updated systematic review. Age Ageing.

[16] Cumming R.G., Thomas M., Szonyi G., Frampton G., Salkeld G., Clemson L. (2001). Adherence to occupational therapist recommendations for home modifications for falls prevention. Am. J. Occup. Ther..

[17] MacCulloch P.A., Gardner T., Bonner A. (2007). Comprehensive fall prevention programs across settings: A review of the literature. Geriatr. Nurs..

[18] Fordham B., Williamson E. (2025). The Role of Social Prescribers in Engaging Older Adults in Strength and Balance Training After Being Discharged From Physiotherapy Rehabilitation: A Qualitative Investigation. Health Soc. Care Community.

[19] Costa A., Henriques J., Alarcão V., Madeira T., Virgolino A., Henriques A., Feteira-Santos R., Polley M., Arriaga M., Nogueira P. (2024). Social prescribing for older adults in mainland Portugal: Perceptions and future prospects. Prev. Med. Rep..

[20] Muhl C., Mulligan K., Bayoumi I., Ashcroft R., Godfrey C. (2023). Establishing internationally accepted conceptual and operational definitions of social prescribing through expert consensus: A Delphi study. BMJ Open.

[21] O’Rourke H.M., Collins L., Sidani S. (2018). Interventions to address social connectedness and loneliness for older adults: A scoping review. BMC Geriatr..

[22] Rogers W.A., Mitzner T.L. (2017). Envisioning the future for older adults: Autonomy, health, well-being, and social connectedness with technology support. Futures.

[23] Høst D., Hendriksen C., Borup I. (2011). Older people’s perception of and coping with falling, and their motivation for fall-prevention programmes. Scand. J. Public Health.

[24] Park Y., Kim S.R., Seo H.-J., Cho J. (2024). Health Literacy in Fall-Prevention Strategy: A Scoping Review. Asian Nurs. Res..

[25] Thomas E., Battaglia G., Patti A., Brusa J., Leonardi V., Palma A., Bellafiore M. (2019). Physical activity programs for balance and fall prevention in elderly: A systematic review. Medicine.

[26] Percival A., Newton C., Mulligan K., Petrella R.J., Ashe M.C. (2022). Systematic review of social prescribing and older adults: Where to from here?. Fam. Med. Community Health.

[27] The EuroQol Group (1990). EuroQol-a new facility for the measurement of health-related quality of life. Health Policy.

[28] Sullivan G.M., Artino A.R. (2013). Analyzing and interpreting data from Likert-type scales. J. Grad. Med. Educ..

[29] Warmoth K., Lang I.A., Phoenix C., Abraham C., Andrew M.K., Hubbard R.E., Tarrant M. (2016). ‘Thinking you’re old and frail’: A qualitative study of frailty in older adults. Ageing Soc..

[30] Alfaro Hudak K.M., Adibah N., Cutroneo E., Liotta M., Sanghera A., Weeks-Gariepy T., Strunz E., Rein D.B. (2023). Older adults’ knowledge and perception of fall risk and prevention: A scoping review. Age Ageing.

[31] Burgon C., Darby J., Pollock K., Van Der Wardt V., Peach T., Beck L., Logan P., Harwood R.H. (2019). Perspectives of healthcare professionals in England on falls interventions for people with dementia: A qualitative interview study. BMJ Open.

[32] Äijö M., Clifford A.M., Maguire I.O. (2023). Preventing falls: Emphasizing education to support older person active ageing. Global Perspectives on Health Assessments for an Aging Population.

[33] Khong L., Farringdon F., Hill K.D., Hill A.-M. (2015). “We are all one together”: Peer educators’ views about falls prevention education for community-dwelling older adults-a qualitative study. BMC Geriatr..

[34] Howarth M., Griffiths A., Da Silva A., Green R. (2020). Social prescribing: A ‘natural’community-based solution. Br. J. Community Nurs..

[35] Prochaska J.O., DiClemente C.C. (1983). Stages and processes of self-change of smoking: Toward an integrative model of change. J. Consult. Clin. Psychol..

[36] Rosenstock I.M. (1974). The health belief model and preventive health behavior. Health Educ. Monogr..

[37] Johnston Y.A., Bergen G., Bauer M., Parker E.M., Wentworth L., McFadden M., Reome C., Garnett M. (2019). Implementation of the stopping elderly accidents, deaths, and injuries initiative in primary care: An outcome evaluation. Gerontologist.

[38] Wee J., Lysaght R. (2009). Factors affecting measures of activities and participation in persons with mobility impairment. Disabil. Rehabil..

[39] Smith M.L., Ory M.G. (2023). Multi-directional nature of falls among older adults: A rationale for prevention and management. Front. Public Health.

[40] Wiseman J.M., Quatman C.E., Quatman-Yates C.C. (2025). Examining Factors Influencing Older Adult Engagement in Fall Prevention: A Comparative Analysis Among Stakeholders. J. Am. Geriatr. Soc..

[41] WHO Team (2020). Healthy Ageing and Functional Ability [Internet].

[42] Chesser A.K., Woods N.K., Reyes J., Rogers N.L. (2018). Health literacy and older adults: Fall prevention and health literacy in a midwestern state. J. Aging Res. Healthc..

[43] Fixsen D., Scott V., Blase K., Naoom S., Wagar L. (2011). When evidence is not enough: The challenge of implementing fall prevention strategies. J. Saf. Res..

[44] Frick K.D., Kung J.Y., Parrish J.M., Narrett M.J. (2010). Evaluating the cost-effectiveness of fall prevention programs that reduce fall-related hip fractures in older adults. J. Am. Geriatr. Soc..

